# Highly contrasted population genetic structures in a host–parasite pair in the Caribbean Sea

**DOI:** 10.1002/ece3.3413

**Published:** 2017-10-04

**Authors:** Quentin Jossart, Chantal De Ridder, Harilaos A. Lessios, Mathieu Bauwens, Sébastien Motreuil, Thierry Rigaud, Rémi A. Wattier, Bruno David

**Affiliations:** ^1^ Département de Biologie des Organismes Laboratoire de Biologie Marine Université Libre de Bruxelles (ULB) Brussels Belgium; ^2^ Biogéosciences UMR CNRS 6282 Université de Bourgogne Franche‐Comté (UBFC) Dijon France; ^3^ Smithsonian Tropical Research Institute Balboa Panama; ^4^ Museum National d'Histoire Naturelle (MNHN) Paris France

**Keywords:** CO1, crab, microsatellites, population genetics, sea urchin

## Abstract

Evolution and population genetic structure of marine species across the Caribbean Sea are shaped by two complex factors: the geological history and the present pattern of marine currents. Characterizing and comparing the genetic structures of codistributed species, such as host–parasite associations, allow discriminating the relative importance of environmental factors and life history traits that influenced gene flow and demographic events. Using microsatellite and Cytochrome Oxidase I markers, we investigated if a host–parasite pair (the heart urchin *Meoma ventricosa* and its parasitic pea crab *Dissodactylus primitivus*) exhibits comparable population genetic structures in the Caribbean Sea and how the observed patterns match connectivity regions from predictive models and other taxa. Highly contrasting patterns were found: the host showed genetic homogeneity across the whole studied area, whereas the parasite displayed significant differentiation at regional and local scales. The genetic diversity of the parasitic crabs (both in microsatellites and COI) was distributed in two main groups, Panama–Jamaica–St Croix on the one hand, and the South‐Eastern Caribbean on the other. At a smaller geographical scale, Panamanian and Jamaican parasite populations were genetically more similar, while more genetic differentiation was found within the Lesser Antilles. Both species showed a signature of population expansion during the Quaternary. Some results match predictive models or data from previous studies (e.g., the Western‐Eastern dichotomy in the parasite) while others do not (e.g., genetic differentiation within the Lesser Antilles). The sharp dissimilarity of genetic structure of these codistributed species outlines the importance of population expansion events and/or contrasted patterns of gene flow. This might be linked to differences in several life history traits such as fecundity (higher for the host), swimming capacity of larval stages (higher for the parasite), and habitat availability (higher for the host).

## 1 INTRODUCTION

**Figure 1:**
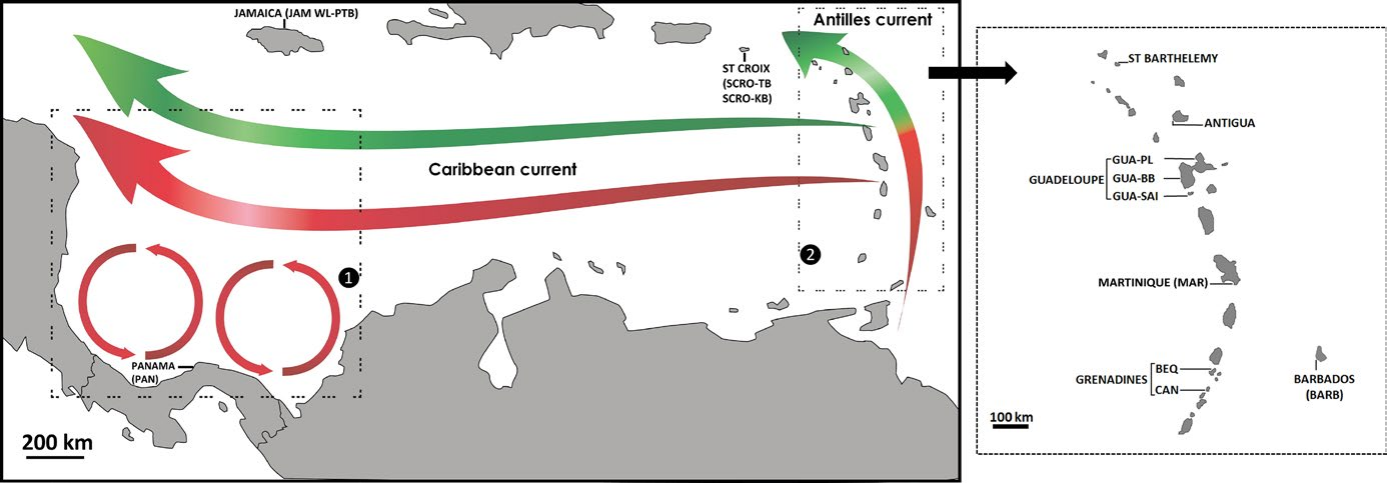
Sampled sites across the Caribbean Sea with schematic pattern of the main currents. Labels 1 and 2, delimited by dashed frames, denote Panama‐Nicaragua and Lesser Antilles “connectivity regions” according to Kool et al. (2010). Colors of the arrows denote differences in current speed (red: >20 cm/s, green: <20 cm/s)

The history and dynamics of marine populations living in the Caribbean Sea have been shaped both by patterns of ocean circulation and geological events. Sea level fluctuations, related to the Quaternary climatic oscillations since 2.5 million years ago (Ma), changed the geography and ecology of the region. Eight climatic cycles have been recorded since 800,000 years ago (Pillans & Gibbard, 2012), notably the last glacial maximum (26 to 21 ka BP, Clark et al., 2009) with a sea level fall of ca. 150 m (Clark et al., 2009; Peltier, 2002; Peltier & Fairbanks, 2006). Such eustatic variations may have affected the distribution and the population genetic structure of extant organisms. The present‐day marine currents are characterized by three main systems namely the “Caribbean current,” the “Antilles current,” and a large eddy from Panama to Costa Rica (Lessios, Robertson, & Cubit, 1984; Gyory, Mariano, & Ryan, 2013; Figure 1). The speed and the direction of these currents (e.g., East to West along the Caribbean current, South to North along the Antilles current) may have implications for the genetic patterns among populations (e.g., direction of gene flow). Integrating the Caribbean marine currents into an oceanographic model, four connectivity regions have been proposed (Cowen, Paris, & Srinivasan, 2006): Eastern Caribbean, Western Caribbean, Bahamas, and Panama‐Colombia (Figure 1). This regional pattern leads to the prediction of high dispersal potential of marine larvae within each region, but limited exchange across them (Cowen et al., 2006). More recently, Kool, Paris, Andréfouët, and Cowen (2010) refined these connectivity regions, sometimes in weak agreement with geographic distances. The three new regions defined by this second model are the Lesser Antilles, Bahamas‐Northern Cuba, and Panama‐Nicaragua. In both models, a break between western and eastern regions is predicted, and Jamaica is suggested as a stepping stone between them. Some genetic studies of Caribbean taxa agree with the separation of these regions (e.g., fish: Purcell, Cowen, Hugues, & Williams, 2006; corals: Foster et al., 2012; and Andras, Rypien, & Harvell, 2013), while others do not, or even reveal no genetic structure at all (e.g., lobster: Silberman, Sarver, & Walsh, 1994; fish: Purcell et al., 2006; gastropod: Diáz‐Ferguson, Haney, Wares, & Silliman, 2010). Additional data are, therefore, needed to further understand the biogeographic regions within the Caribbean Sea.

Comparing the genetic structures of codistributed species can disentangle the relative importance of common history, present‐day ecology, and life history traits on their evolutionary history (e.g., Criscione, 2008; Kool et al., 2010). Parasite–host pairs are necessarily codistributed species with the distribution of parasites overlapping that of their specific habitat (the hosts). This is even more constrained if the set of host species is limited for a given parasite species (Poulin, 2007). Characterizing and comparing the genetic structures of such codistributed species allow the identification of geographical barriers to dispersal (e.g., DeBiasse, Richards, Shivji, & Hellberg, 2016) or clarifies the contribution of landscape fragmentation to their phylogeographies (Rodelo‐Urrego et al., 2013). Moreover, when interacting species have contrasted life histories, their comparison can also reveal which life history traits predominantly affect dispersal and population size among populations despite the shared environment (Criscione, 2008; Kochzius et al., 2009), and ultimately the co‐evolutionary history of a given host–parasite association (Du Toit, Van Vuuren, Matthee, & Matthee, 2013). Finally, host–parasite costructure studies may help predict the potential for local adaptation by determining the relative dispersal rate between a host and its parasite (Greischar & Koskella, 2007).

Here, we aim to understand how populations of a marine host–parasite pair are genetically structured in the Caribbean. We studied the irregular sea urchin *Meoma ventricosa* and its parasitic pinnotherid crab *Dissodactylus primitivus* (De Bruyn, Rigaud, David, & De Ridder, 2009; Telford, 1982). Both species are endemic to the Caribbean Sea and to neighboring American coasts, from Florida down to Brazil (Alvarado, 2011; Chesher, 1969; Wirtz, de Melo, & De Grave, 2009). *Meoma ventricosa* lives at depths of 1–200 m on soft substrates ranging from small coral pebbles to sandy or fine sediments (Chesher, 1969). *Dissodactylus primitivus* is an ectoparasite of *M. ventricosa* on which it reproduces, finds a shelter, and feeds (Telford, 1982). Prevalence of parasitism is high, with 75%–100% of the sea urchins infected by 1–21 crabs (De Bruyn et al., 2009 and unpublished data). The crab consumes host tegument and spines (Telford, 1982), which induce wounds on the sea urchin (De Bruyn et al., 2009). The association is rather obligatory (the adult crabs live both on the sea urchin body and in the sediment just beneath, De Bruyn et al., 2016), it is specific (only two host species are used, and *M. ventricosa* is the only host harboring juvenile crabs, De Bruyn, David, De Ridder, & Rigaud, 2010) and nonpermanent (larval stages are free). Both *M. ventricosa* and *D. primitivus* have pelagic larvae and therefore are prone to dispersal by marine currents (Emlet, McEdward, & Strathmann, 1987; Pohle & Telford, 1983). However, the respective abundances and swimming abilities of the planktotrophic larvae of pinnotherid crabs and of sea urchins differ sharply: (1) Crab fecundity is thousands of times weaker than that of the sea urchin which might result in a lower dispersal and a lower population expansion capacity (Emlet et al., 1987; Jossart et al., 2014), (2) crab larvae are known to be better “swimmers” which should decrease drifting by marine currents (Metaxas, 2013; Yednock & Neigel, 2011), (3) habitat suitability (the sea urchin's body) is smaller for the parasite which should decrease recruitment rate. These differences in life history traits could cause incongruence in the population genetic patterns of these two partners.

We investigated the genetic variation of this host–parasite association to determine how past and recent ecological contexts shape the population structure of *M. ventricosa* and *D. primitivus*. Using partial sequences of Cytochrome Oxidase subunit I (COI) and microsatellites, we addressed the following questions: (1) Do host and parasite exhibit comparable genetic structures? (2) Do these structures correspond to the connectivity regions from predictive models in the Caribbean area? (3) What are the respective contributions of Quaternary sea levels fluctuations (Clark et al., 2009), of present pattern of marine currents, and of differences in life‐history traits in explaining the crab and sea urchin demographic history or gene flow patterns?

## 2 MATERIALS AND METHODS

### 2.1 Collections

We sampled crabs and sea urchins between 2006 and 2013 at 14 sites (13 for sea urchins) (Table 1; Figure 1). These sites were situated at the Lesser Antilles (St Croix, Saint Barthélemy, Antigua, Guadeloupe, Martinique, Bequia, Canouan, Barbados), Greater Antilles (Jamaica), or Central America (Panama) (Figure 1).

**Table 1:** Sampling information including island/country, site, GPS coordinates, depth, year, and total number of samples for COI and microsatellite (SSR) analyzes

Island/country	Site	Coordinates	Depth (m)	Year	No. of individuals			
					*D. p*		*M. v*	
					SSR	COI	SSR	COI
Panama	Isla Drake (PAN)	9°33′40″N/79°41′2″W	9–22	2013	20	17	17	16
Jamaica	Western Lagoon (JAM‐WL)	18°28′3″N/77°24′42″W	2–4	2006, 2009^a^	30	22	12	13
	Pear Tree Bottom (JAM‐PTB)	18°27′48″N/77°21′14″W	12–18	2009	30	20	—	—
St Croix	Kings Bay (SCRO‐KB)	17°39′59″N/64°48′56″W	8–9	2011	30	24	29	28
	Teague Bay (SCRO‐TB)	17°46′4″N/64°37′59″W	1–3	2011	30	22	26	23
Saint Barthélemy	Anse de Grand Cul de Sac (SBAR)	17°54′39″N/62°48′5″W	1–2	2011	30	20	30	25
Antigua	Middle Reef (ANT)	17°0′23″N/61°51′29″W	2–11	2011	30	23	26	26
Guadeloupe	Port‐Louis (GUA‐PL)	16°25′10″N/61°32′31″W	11	2011	30	23	25	23
	Baie de Bouillante (GUA‐BB)	16°7′52″N/61°46′47″W	6–8	2011	30	26	27	25
	Les Saintes (GUA‐SAI)	15°51′56″N/61°36′0″W	10–17	2011	30	21	25	22
Martinique	Point Borgnèse (MAR)	14°26′18″N/60°54′54″W	10–15	2010	30	21	20	18
Bequia	Lower Bay (BEQ)	12°59′50″N/61°14′51″W	7–9	2011	30	23	30	28
Canouan	Rameau Bay (CAN)	12°43′28″N/61°19′58″W	7–8	2011	30	21	30	23
Barbados	Carlisle Bay (BARB)	13°4′26″N/59°37′0″W	12–15	2012	30	25	30	27
					410	308	327	297

*D. p*,* Dissodactylus primitivus*;* M. v*,* Meoma ventricosa*.

a *D. p* was sampled in 2009 and *M. v* in 2006. Site abbreviations shown here are used in other tables and figures.

Samples were collected by SCUBA diving or snorkeling at depths ranging from 1 to 22 m (Table 1). Sea urchins were collected individually in plastic bags that were immediately tied up after collection. Immediately after the dive, a sample of each sea urchin (3–4 spines) and all the crabs captured on each host were isolated, labeled, and preserved in pure ethanol.

The total numbers of specimens used for microsatellite analyzes were 327 sea urchins and 410 crabs (Table 1). For COI analyzes, we sequenced a total of 297 sea urchins and 309 crabs (Table 1).

### 2.2 DNA extraction

We extracted DNA from one pereiopod of each crab using the Chelex resin method (see the detailed protocol in Jossart et al., 2014) and from two spines of each sea urchin using the Qiagen DNeasy Blood & Tissue Kit.

### 2.3 COI data collection and analysis

For crabs, we amplified a 652 base pair fragment using the primers LCO1490 (5′‐GGTCAACAAATCATAAAGATATTGG‐3′) and HCO2198 (5′‐TAAACTTCAGGGTGACCAAAAAATCA‐3′) (Folmer, Black, Hoeh, Lutz, & Vrijenhoek, 1994). Each PCR included 7.5 μl of Master Mix Qiagen (Taq Polymerase, nucleotides), 2 μl of DNA, 0.6 μl (10 μmol/L) of each forward or reverse primer and 4.3 μl of sterile water. PCR conditions consisted of 35 cycles for each of the three temperature steps [60 s at 94°C (denaturation), 60 s at 40°C (annealing), and 120 s at 72°C (elongation)]. These cycles were preceded by a step of 2 min at 94°C and were followed by a step of 2 min at 72°C. After amplification, 0.8 μl of sterile water was added, with 0.2 μl (10 units/μl) of Exonuclease I (Affymetrix) and 1 μl (1 unit/μl) of Shrimp Alkaline Phosphatase (Affymetrix), to purify amplified DNA from dNTPs and primers. Samples were incubated for 60 min at 37°C, and 10 min at 80°C. The samples were then dried overnight in an oven at 37°C. Finally, plates containing the samples were sent to the MACROGEN sequencing service. Sequence editing and alignment were performed using MEGA 5.1 (Tamura et al., 2011).

For sea urchins, we amplified a 758 base pair fragment of COI using the primers characterized by Stockley, Smith, Littlewood, Lessios, & Mackenzie‐Dodds, 2005 (5′‐GCYTGAGCWGGCATGGTAGG‐3′/5′‐GCTCGTGCRTCTACRTCCAT‐3′). Each PCR (15 μl) included 7.5 μl of Master Mix Qiagen (Taq Polymerase, nucleotides), 1 μl of DNA, 0.3 μl (10 μmol/L) of each forward or reverse primer, and 5.9 μl of sterile water. PCR conditions consisted of 35 cycles for each of the three temperature steps [40 s at 94°C (denaturation), 30 s at 52°C (annealing) and 60 s at 72°C (elongation)]. These cycles were preceded by a step of 4 min at 95°C and were followed by a step of 5 min at 72°C. Purification and sequencing steps were identical to those for the crabs.

We used Arlequin 3.5 (Excoffier & Lischer, 2010) to calculate the number of haplotypes (*N*_a_), the effective number of haplotypes (*N*_e_), the haplotype diversity (*h*), the mean pairwise differences among haplotypes (MPD), and the nucleotide diversity (π). Haplotype networks (Minimum Spanning Networks) were constructed using MINSPNET (Excoffier & Smouse, 1994) and HapStar 0.5 (Teacher & Griffiths, 2011).

We evaluated pairwise differentiation between populations from different locations in four ways: Φ_ST_ (Hudson, Slatkin, & Maddison, 1992 based on Tajima and Nei (1984) genetic distances, conventional *F*_ST_ (Weir & Cockerham, 1984), exact tests for population differentiation with Arlequin 3.5 (significance evaluated using 10,000 permutations for *F*_ST_ statistics and 100,000 permutations for exact tests, Goudet, Raymond, de Meeüs, & Rousset, 1996), and Jost's D (Jost, 2008) using SPADE (bootstrap replicates of 10,000) (Chao & Shen, 2010).

Using SAMOVA 2.0, we performed (for crabs) a Spatial Analysis of MOlecular VAriance (SAMOVA, Dupanloup, Schneider, & Excoffier, 2013). We calculated Φ_CT_ for seven possible groupings (from 2 to 8) in order to find the grouping that maximizes the genetic variance among groups. Analysis of molecular variance (AMOVA, Excoffier, Smouse, & Quattro, 1992) was performed with Arlequin 3.5 (significance of Φ values was determined by a permutation test of 10,000 randomizations). Regions for AMOVA were defined according to the SAMOVA analysis (see results): Region 1 (Panama, Jamaica, St Croix, Saint Barthélemy, Antigua, and Guadeloupe), Region 2 (Martinique, Bequia, Canouan, and Barbados).

We tested isolation by distance (IBD) with a Mantel test (Φ_ST_ vs. km), using the software Mantel 1.19 (life.bio.sunysb.edu/morph/soft‐mult.html). The geographical distance corresponded to the shortest distance avoiding islands/strips of land and was calculated using the path tool in Google Earth. IBD was performed for the whole dataset and inside the “Lesser Antilles” connectivity region (excluding St Croix) defined by Kool et al. (2010) (see Figure 1).

We used three methods to verify the existence of population expansion. For these analyzes, locations showing no differentiation in other analyzes were pooled (see Section 2.1). First, using Arlequin 3.5, we calculated the following: (1) Fu's *F*_S_ statistic, testing for an excess of recently evolved haplotypes in an expanding population compared with a stable population (Fu, 1997). The significance of the *F*_S_ was determined by a permutation test using 10,000 randomizations. (2) The sum of squared deviation (SSD) between the observed distribution of the number of nucleotide differences and the unimodal mismatch distribution expected from population expansion (Rogers, Fraley, Bamshad, Watkins, & Jorde, 1996; Schneider & Excoffier, 1999). SSD was also calculated to evaluate a potential spatial (range) expansion (Ray, Currat, & Excoffier, 2003). The significance of the observed mismatch was verified by a test of goodness‐of‐fit (10,000 bootstraps). For the spatial expansion analysis, we also estimated the time of expansion (Schenekar & Weiss, 2011). (3) Past changes in effective population size were evaluated using the Extended Bayesian Skyline Plot approach (EBSP) in BEAST 2.4.4 (Bouckaert et al., 2014). For crabs, accurate estimates were not possible, because populations were differentiated and could not be pooled. For sea urchins, BEAST was performed for 2.10^7^ iterations (10% of burnin), using a pairwise divergence rate of 1.52% per million years (Lessios, 2008) and HKY as the substitution model. Trace file was checked (including ESS values always > 200) using Tracer 1.6. The skyline plot was performed with an R script developed by the BEAST authors.

### 2.4 Microsatellite data collection and analysis

For crabs, we used ten loci already used for studying Jamaican populations (Jossart et al., 2013, 2014). These loci were multiplex amplified and genotyped with an AB 3730 DNA Analyzer (see Jossart et al., 2013 for detailed protocol and primer sequences).

For *M. ventricosa*, we used eight microsatellite loci (Jossart, Geyer, & Lessios, 2015). Microsatellites were amplified in simplex according to the tagged primer‐method and genotyped in an AB 3130XL Genetic Analyzer (see Jossart et al., 2015 for detailed protocol and primer sequences). We evaluated (using POWSIM 4.1, Ryman & Palm, 2006) that these microsatellites had a statistical power (1–β) of 0.999 (associated with an *F*_ST_ of 0.0075) for the present dataset. The retained parameter values were selected according to the instructions of POWSIM manual (Ne of 2000; 10 generations of drift; 1,000 runs).

Using Genepop 4.2.2, the frequency of assumed null alleles was calculated, and linkage disequilibrium was tested for each locus pair within each species (Rousset, 2008). We used the software FSTAT 2.9.3.2 (Goudet, 1995) to estimate number of alleles and allelic richness (AR). Differences between sites in mean AR were tested using a Kruskal–Wallis test. We assessed deviations from Hardy–Weinberg equilibrium (*F*_IS_) using FSTAT. The significance of *F*_IS_ was evaluated for each species using permutation tests: one testing for heterozygote excess and the other testing for heterozygote deficiency.

We calculated pairwise *F*_ST_ (Weir and Cockerham's Theta, θ_WC_) between different populations using SPAGeDi 1.4 (Hardy & Vekemans, 2002; Weir & Cockerham, 1984). The significance of *F*_ST_ was evaluated using a permutation test (20,000 permutations). For *M. ventricosa*, we also estimated *F*_ST_ values adjusted for null alleles with the software FreeNA (Chapuis & Estoup, 2007). Using Genepop 4.2.2, we performed pairwise exact tests of differentiation between populations (Goudet et al., 1996). Because *F*_ST_ values are sensitive to the marker's heterozygosity, we also calculated pairwise Jost's (2008) using the software DEMEtics (Gerlach, Jueterbock, Kraemer, Deppermann, & Harmand, 2010). We analyzed the molecular variance (AMOVA, with the same regions as the AMOVA for COI) with GenAlEx 6.502 (Peakall & Smouse, 2012). Significance of *F* values was determined by a permutation test (10,000 randomizations). We evaluated the possibility of isolation by distance (IBD) by a Mantel test (*F*_ST_ vs. km) using the software Mantel 1.19. IBD was performed for the whole dataset and inside the “Lesser Antilles” connectivity region (excluding St Croix) defined by Kool et al. (2010) (see Figure 1).

To infer the most probable number of genetic clusters (K), we used STRUCTURE 2.3.4 (Pritchard, Stephens, & Donnelly, 2000). For *M. ventricosa*, we assumed values of *K* between 1 and 13 and 10 independent simulations, using the following parameters: running chain lengths of 100,000, admixture model (indicating the sampling location to the software from the results of *F*_ST_, *D* and exact tests), alpha inferred, allele frequencies correlated among populations, and possibility of null alleles. For *D. primitivus*, we used STRUCTURE with values of *K* between 1 and 14 and 10 independent simulations, using the following parameters: running chain lengths of 100,000, admixture model (without entering the sampling location in the analysis), alpha inferred, and allele frequencies correlated among populations. We determined the most likely value of *K* using the original method (described in the STRUCTURE manual) and the method of Evanno, Regnaut, and Goudet (2005) implemented in STRUCTURE HARVESTER (Earl & vonHoldt, 2012). Bar plots were created using the software DISTRUCT 1.1 (Rosenberg, 2004). In order to confirm STRUCTURE's assignments to genetic clusters in *D. primitivus*, we used BAPS 6.0 (Corander & Marttinen, 2006).

We also used the software divMigrate (Sundqvist, Keenan, Zackrisson, Prodöhl, & Kleinhans, 2016) to detect potential asymmetric gene flow between pairs of populations. For this analysis, the undifferentiated sites from the same island were pooled together. Significance of asymmetry (10,000 bootstraps) was assed using the tool implemented in the software.

## 3 RESULTS

### 3.1 COI data

**Figure 2:**
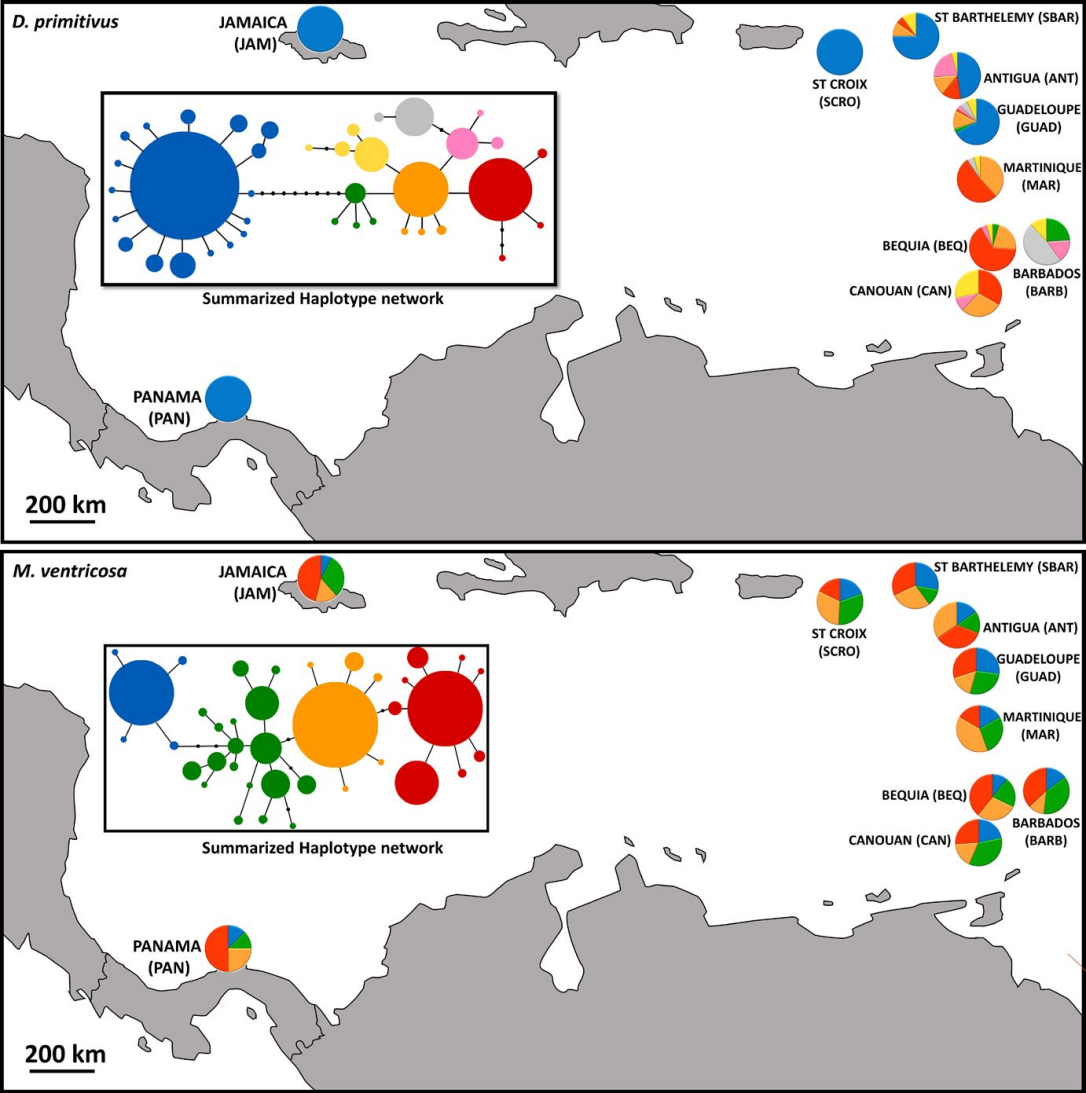
Summarized COI haplotype network (internal frame; colors denote each group of haplotypes) in *Dissodactylus primitivus* (top) and *Meoma ventricosa* (bottom). Pie charts in the main frame represent the proportion of haplotypes at each location

In the crab *D. primitivus*, we found 39 haplotypes in 308 sequenced individuals. They were distributed in two divergent clades (18 haplotypes in clade A vs. 21 haplotypes in clade B) separated by 10 substitutions (Figure 2, Appendix S1). Panama, Jamaica, and Saint Croix harbored crabs with haplotypes exclusively from the A group while crabs from Martinique, Bequia, Canouan, and Barbados all belonged to B group. The remaining islands (Guadeloupe, Antigua, and Saint Barthélemy) harbored crabs with haplotypes from both groups. The mean number of haplotypes per site was 6.21 (±1.76), the mean effective number of haplotypes was 3.02 (±1.46), and the haplotype diversity was 0.63 (±0.18, Table 2). The mean nucleotide diversity was moderate (0.0043 ± 0.0038), and the mean pairwise differences (MPD) among haplotypes within a given site were 2.82 (±2.49), with low values for islands that contained only one of the haplotype groups (Table 2).

**Table 2:** Diversity indices for COI data in *Dissodactylus primitivus* and *Meoma ventricosa*. *N*, sample size; *N*_a_, number of haplotypes; *N*_e_, effective number of haplotypes; *h*, haplotype diversity; MPD, mean pairwise substitutions among haplotypes; π, nucleotide diversity. See Table 1 for site abbreviations

	*Dissodactylus primitivus*						*Meoma ventricosa*					
	*N*	*N*_a_	*N*_e_	*h*	MPD	Π (%)	*N*	*N*_a_	*N*_e_	*h*	MPD	Π (%)
PAN	17	4	2.39	0.62 ± 0.11	0.84 ± 0.63	0.13 ± 0.11	16	7	4.92	0.85 ± 0.06	2.77 ± 1.54	0.37 ± 0.23
JAM‐WL	22	6	2.92	0.69 ± 0.10	1.00 ± 0.70	0.15 ± 0.12	13	9	5.54	0.94 ± 0.05	3.86 ± 2.07	0.51 ± 0.31
JAM‐PTB	20	8	3.45	0.75 ± 0.10	1.12 ± 0.76	0.17 ± 0.13	—	—	—	—	—	—
SCRO‐TB	24	2	1.31	0.25 ± 0.11	0.25 ± 0.29	0.03 ± 0.05	28	9	5.04	0.84 ± 0.05	2.85 ± 1.56	0.38 ± 0.23
SCRO‐KB	22	4	1.42	0.31 ± 0.12	0.33 ± 0.34	0.05 ± 0.05	23	13	7.84	0.90 ± 0.04	3.39 ± 1.79	0.48 ± 0.26
SBAR	20	8	2.60	0.65 ± 0.12	5.03 ± 2.55	0.77 ± 0.44	25	9	5.08	0.84 ± 0.05	2.99 ± 1.61	0.39 ± 0.24
ANT	23	8	6.87	0.89 ± 0.03	7.04 ± 3.43	1.080 ± 0.59	26	9	5.12	0.84 ± 0.05	3.06 ± 1.65	0.40 ± 0.24
GUAD‐PL	23	7	2.74	0.66 ± 0.10	6.11 ± 3.02	0.94 ± 0.52	23	10	5.45	0.85 ± 0.06	3.25 ± 1.74	0.43 ± 0.26
GUAD‐BB	26	7	1.83	0.47 ± 0.12	4.73 ± 2.39	0.73 ± 0.41	25	11	6.87	0.89 ± 0.04	3.08 ± 1.66	0.41 ± 0.24
GUAD‐SAI	21	6	2.41	0.61 ± 0.12	6.50 ± 3.20	1.000 ± 0.55	22	10	5.76	0.87 ± 0.05	3.43 ± 1.82	0.45 ± 0.27
MAR	21	6	2.96	0.70 ± 0.07	1.37 ± 0.88	0.21 ± 0.15	18	9	5.59	0.87 ± 0.06	3.05 ± 1.67	0.40 ± 0.25
BEQ	23	7	2.44	0.62 ± 0.11	1.17 ± 0.78	0.18 ± 0.13	28	17	13.07	0.96 ± 0.02	3.66 ± 1.91	0.48 ± 0.28
CAN	21	7	4.74	0.83 ± 0.05	1.50 ± 0.94	0.23 ± 0.16	23	11	7.45	0.91 ± 0.03	3.46 ± 1.83	0.46 ± 0.27
BARB	25	7	4.25	0.80 ± 0.06	2.43 ± 1.36	0.37 ± 0.23	27	12	6.57	0.88 ± 0.04	3.20 ± 1.70	0.42 ± 0.25
Average	22 ± 2.29	6.21 ± 1.76	3.02 ± 1.46	0.63 ± 0.18	2.82 ± 2.49	0.43 ± 0.38	23 ± 4.63	10.46 ± 2.50	6.55 ± 2.12	0.88 ± 0.04	3.23 ± 0.32	0.43 ± 0.04

In the sea urchin *M. ventricosa*, the total number of haplotypes was 38 of a total of 297 individuals sequenced. There were three haplotypes represented in high frequency (H1 20%, H2 18% and H3 19%; Figure 2, Appendix S2). The mean MPD among haplotypes across sampling sites was equal to 3.23 (±0.32) with a narrow range from 2.77 to 3.86, the mean number of haplotypes per locality was 10.46 (±2.50), the mean effective number of haplotypes was 6.55 (±2.12), the nucleotide diversity was moderate (0.0043 ± 0.0004), and the mean haplotype diversity was high 0.88 (±0.04) in all sites (Table 2).

Φ_ST_ values between populations were highly different between the host and its parasite (Table 3). None was significantly different from 0 for *M. ventricosa*, while most were significant and large for *D. primitivus* (Table 3). All Φ_ST_ pairwise comparisons in *D. primitivus* involving Martinique, Bequia, Canouan, and Barbados were significant and some of them were close to 1 (Table 3). Jost's *D* and exact tests of differentiation and conventional *F*_ST_ indicated the same trends as Φ_ST_ except for some comparisons with islands harboring haplotypes from both haplotype groups (Appendix S8). For *D. primitivus*, SAMOVA analysis showed a maximum Φ_CT_ value for a population structure of two groups (Φ_CT_ = 0.666, *p* < .001). Group 1 included Panama, Jamaica, St Croix, Saint Barthélemy, Antigua, and Guadeloupe, and group 2 was composed of Martinique, Bequia, Canouan, and Barbados. Φ_CT_ values for other possible patterns of population structure were close (e.g., 0.656 for *K* = 3 and 0.646 for *K* = 4). *K* = 3 is associated with the segregation of Barbados and *K* = 4 with the segregation of Antigua. Φ_CT_ values from AMOVA (among regions) were equal to 0.666 (*p* < .001) for *D. primitivus* and 0 for *M. ventricosa* (*p* = .53) (Appendix S9).

**Table 3:** Pairwise ΦST values (based on Tajima & Nei, 1984 distances) between populations for COI analysis in *Dissodactylus primitivus* (above diagonal) and *Meoma ventricosa* (below diagonal). Bold values differ significantly from zero (based on 10,000 permutations). The alpha value was corrected by Benjamini–Yekutieli method (Narum, 2006) and were 0.0094 for *D. primitivus* and 0.0099 for *M. ventricosa*. See Table 1 for site abbreviations

	PAN	JAM‐WL	JAM‐PTB	SCRO‐TB	SCRO‐KB	SBAR	ANT	GUAD‐PL	GUAD‐BB	GUAD‐SAI	MAR	BEQ	CAN	BARB
PAN		−0.022	0.022	**0.12**	**0.1**	0.159	**0.395**	**0.238**	0.169	**0.266**	**0.907**	**0.916**	**0.899**	**0.863**
JAM‐WL	−0.017		−0.023	**0.089**	**0.075**	**0.17**	**0.415**	**0.254**	**0.179**	**0.283**	**0.903**	**0.911**	**0.896**	**0.865**
JAM‐PTB	—	—		**0.08**	**0.067**	0.158	**0.401**	**0.238**	**0.168**	**0.266**	**0.898**	**0.906**	**0.891**	**0.858**
SCRO‐TB	0.022	0.025	—		−0.022	**0.194**	**0.443**	**0.279**	**0.2**	**0.311**	**0.934**	**0.94**	**0.927**	**0.89**
SCRO‐KB	0.063	0.028	—	−0.016		**0.198**	**0.451**	**0.285**	**0.204**	**0.318**	**0.932**	**0.938**	**0.926**	**0.891**
SBAR	−0.007	0.017	—	−0.011	0.01		0.093	−0.017	−0.042	−0.009	**0.663**	**0.681**	**0.648**	**0.649**
ANT	−0.019	0.008	—	−0.012	0.022	−0.02		0.023	0.093	0.007	**0.367**	**0.389**	**0.347**	**0.382**
GUAD‐PL	0.001	−0.019	—	0.044	0.062	0.034	0.025		−0.018	−0.041	**0.552**	**0.573**	**0.534**	**0.534**
GUAD‐BB	0.026	0.025	—	−0.022	−0.013	−0.024	0.002	0.031		−0.01	**0.651**	**0.667**	**0.637**	**0.639**
GUAD‐SAI	0.034	0.04	—	0.004	0.004	−0.019	0.013	0.038	−0.027		**0.524**	**0.547**	**0.503**	**0.509**
MAR	0.032	0.047	—	−0.028	−0.009	−0.003	−0.018	0.059	−0.006	−0.001		−0.014	0.032	**0.337**
BEQ	−0.028	−0.027	—	−0.007	0.019	−0.01	−0.022	0.005	0.002	0.018	0,000		0.101	**0.402**
CAN	0.01	0.003	—	−0.012	0.001	−0.004	0.002	−0.005	−0.019	−0.018	−0.009	−0.006		**0.3**
BARB	−0.002	−0.018	—	0.018	0.032	0.016	0.013	−0.018	0.013	0.019	0.028	−0.009	−0.023	

The Mantel test for crabs (Φ_ST_ vs. km) showed a correlation between genetic and geographic distances, indicative of isolation by distance for the whole dataset (*r* = .262, *p* < .04) and when only the Lesser Antilles were considered (*r* = .524; *p* < .002) (Appendix S10). All the Mantel tests for sea urchins were not significant (whole dataset: *r* = .161, *p* = .15; Lesser Antilles only: *r* = −0.192, *p* = .86).

In *D. primitivus* (seven groups), Fu's *F*_S_ was negative and significant for two groups (Jamaica, Martinique + Bequia + Canouan), and mismatch analyzes did not reject the null hypothesis of pure demographic expansion nor a spatial expansion for all groups (see Appendix S6). Assuming a pairwise divergence rate of 2% per million years for COI (data of several crustaceans from Lessios, 2008), the time of spatial expansion varied between 25,503 years for St Croix (90% CI: 7,561–66,323) and 919,688 years for St Barthélemy (27,544–6,930,094) with an average time of 328, 336 years (44,196–1,322,777).

In *M. ventricosa* (one group, see Appendix S6), Fu's *F*_S_ was negative and significant (−18.49, *p* = .0008). Mismatch analyzes rejected the null hypothesis of pure demographic expansion (SSD = 0.0331, *p* = .012) but did not reject the spatial expansion null hypothesis (SSD = 0.0275, *p* = .066). Assuming a divergence rate of 1.52% per million years for COI (data of *Meoma* from Lessios, 2008) the time of spatial expansion was evaluated to 341,509 years (90% CI: 164,455–459,155). Extended Bayesian Skyline Plot analysis suggested an increase of population size from around 100,000 years ago (Appendix S7).

### 3.2 Microsatellite data

In both *D. primitivus* and *M. ventricosa*, no linkage disequilibrium was detected between pairs of loci in each population (600 and 364 pairwise comparisons, alpha was Benjamini–Yekutieli corrected to 0.0071 and 0.0076, respectively) (Narum, 2006). For *D. primitivus*, the frequencies of null alleles were low (<0.10) for each locus for the large majority (96%) of sampling sites. Conversely, two loci in *M. ventricosa* (NLQK, 6SKB) had null allele frequencies higher than 0.10 in most of the sites. In *D. primitivus*, there was no heterozygote deficiency in any population, whereas five populations showed heterozygote excess (Table 4, Appendix S3). In *M. ventricosa*, most of the sites showed heterozygote deficiency that can be linked to the presence of null alleles in NLQK and 6SKB (Table 4, Appendix S4). The average number of alleles was 8.6 (±1.0) in *D. primitivus* and 7.8 (±0.6) in *M. ventricosa*. In *D. primitivus*, the mean Allelic Richness (AR) was 7.2 (±0.7) and did not significantly differ among sites (Kruskal‐Wallis test: *H* = 10.00; *p* = .69). In *M. ventricosa*, the mean AR was 6.3 (±0.2) and did not significantly differ among sites (Kruskal‐Wallis test: *H* = 0.97; *p* = 1).

**Table 4:** Diversity indices for microsatellite data for *Dissodactylus primitivus* and *Meoma ventricosa*. Number of individuals (*N*), number of alleles (*N*_A_), allelic Richness (AR), and *F*_IS_ values for microsatellite data in *D. primitivus* and *M. ventricosa*

	*Dissodactylus primitivus*				*Meoma ventricosa*			
	*N*	*N*_A_	AR	*F*_IS_	*N*	*N*_A_	AR	*F*_IS_
PAN	20	7.0 ± 1.2	6.5 ± 1.3	−0.104*	17	7.0 ± 3.9	6.1 ± 3.0	0.122**
JAM‐WL	30	7.8 ± 2.0	6.7 ± 1.7	−0.076*	12	6.9 ± 4.0	6.7 ± 3.9	0.090
JAM‐PTB	30	8.5 ± 1.9	7.4 ± 1.6	−0.127*	—	—	—	—
SCRO‐TB	30	9.7 ± 3.6	7.7 ± 2.6	0.025	29	8.0 ± 5.0	6.2 ± 3.3	0.165**
SCRO‐KB	30	9.9 ± 4.0	8.0 ± 2.8	0.059	26	8.1 ± 4.8	6.3 ± 3.2	0.140**
SBAR	30	9.1 ± 2.6	8.0 ± 2.2	−0.098*	30	8.8 ± 5.3	6.6 ± 3.5	0.145**
ANT	30	8.3 ± 2.8	7.1 ± 2.3	−0.077*	26	7.3 ± 4.3	6.0 ± 3.1	0.040
GUAD‐PL	30	9.2 ± 2.9	7.5 ± 2.2	0.034	25	8.6 ± 4.7	6.6 ± 3.2	0.150**
GUAD‐BB	30	8.5 ± 2.6	7.3 ± 2.3	0.021	27	7.6 ± 3.9	6.2 ± 2.9	0.061
GUAD‐SAI	30	9.4 ± 2.5	7.8 ± 2.2	−0.006	25	7.9 ± 4.4	6.0 ± 3.0	0.113**
MAR	30	9.1 ± 2.8	7.3 ± 2.5	−0.007	20	7.4 ± 3.5	6.3 ± 2.8	0.092
BEQ	30	9.3 ± 3.1	7.5 ± 2.2	−0.030	30	7.9 ± 3.9	6.3 ± 3.0	0.198**
CAN	30	8.3 ± 2.6	7.0 ± 2.1	0.025	30	7.9 ± 4.8	6.2 ± 3.1	0.168**
BARB	30	6.7 ± 3.1	5.6 ± 2.4	−0.049	30	8.0 ± 4.3	6.2 ± 3.0	0.205**
Mean	29 ± 2.67	8.6 ± 1.0	7.2 ± 0.7	−0.029 ± 0.059	25 ± 5.63	7.8 ± 0.6	6.3 ± 0.2	0.130 ± 0.050

*Indicates heterozygote excess (*p* < .01, Benjamini–Yekutieli corrected), and ** indicates heterozygote deficiency (*p* < .01, Benjamini–Yekutieli corrected). See Table 1 for site abbreviations.

*F*_*ST*_ results of the microsatellites were different between the two species (Table 5). The large majority of *F*_ST_ values were close to 0 for *M. ventricosa* regardless of whether they were adjusted for null alleles or not (Table 5). Only three were significantly different from zero, but the values were very small (Table 5). In *D. primitivus,* most *F*_ST_ pairwise values comparing populations of different locations were significantly different from 0, while *F*_ST_ pairwise values among populations of the same island were not significantly different from 0 (Table 5). The highest observed values of *F*_ST_ were among populations from Barbados and those from other localities. Jost's *D* values and exact tests of differentiation led to the same trends (results not shown). *F*_CT_ values from AMOVA (among regions) were equal to 0.026 (*p* < .0001) for *D. primitivus* and 0.00001 for *M. ventricosa* (*p* = .42) (Appendix S9).

**Table 5:** *F*_ST_ values for microsatellite analysis in *Dissodactylus primitivus* (above diagonal) and *Meoma ventricosa* (below diagonal). Bold values differ significantly from zero (20,000 permutations). Alpha was corrected by Benjamini–Yekutieli method and was 0.0094 for *D. primitivus* and 0.0099 for *M. ventricosa*. See Table 1 for site abbreviations

	PAN	JAM‐WL	JAM‐PTB	SCRO‐TB	SCRO‐KB	SBAR	ANT	GUAD‐PL	GUAD‐BB	GUAD‐SAI	MAR	BEQ	CAN	BARB
PAN		**0.0159**	**0.0196**	**0.0379**	**0.0342**	**0.0478**	**0.0658**	**0.0628**	**0.0745**	**0.0754**	**0.0807**	**0.0721**	**0.0926**	**0.1086**
JAM‐WL	0.0014		0.0056	**0.0258**	**0.0266**	**0.0393**	**0.0613**	**0.0480**	**0.0523**	**0.0491**	**0.0671**	**0.0555**	**0.0742**	**0.0950**
JAM‐PTB	—	—		**0.0273**	**0.0235**	**0.0327**	**0.0567**	**0.0503**	**0.0495**	**0.0540**	**0.0677**	**0.0583**	**0.0675**	**0.0949**
SCRO‐TB	0.0020	−0.0083	—		−**0.0013**	**0.0154**	**0.0332**	**0.0145**	**0.0229**	**0.0224**	**0.0471**	**0.0302**	**0.0509**	**0.0790**
SCRO‐KB	0.0114	0.0024	—	−0.0021		**0.0205**	**0.0375**	**0.0202**	**0.0246**	**0.0285**	**0.0526**	**0.0400**	**0.0601**	**0.0947**
SBAR	−0.0019	0.0064	—	0.0001	0.0030		**0.0214**	**0.0212**	**0.0207**	**0.0211**	**0.0391**	**0.0251**	**0.0363**	**0.0654**
ANT	0.0124	−0.0024	—	0.0018	−0.0015	0.0011		0.0186	0.0222	**0.0253**	**0.0347**	**0.0229**	**0.0349**	**0.0715**
GUAD‐PL	0.0050	0.0038	—	−0.0076	−0.0094	−0.0069	−0.0076		−0.0019	−0.0005	**0.0286**	**0.0198**	**0.0240**	**0.0636**
GUAD‐BB	0.0115	0.0091	—	0.0062	−0.0071	0.0037	−0.0035	−0.0051		−0.0015	**0.0194**	0.0100	**0.0158**	**0.0618**
GUAD‐SAI	−0.0053	−0.0056	—	0.0042	0.0094	0.0045	0.0076	0.0011	**0.0187**		**0.0328**	**0.0195**	**0.0291**	**0.0701**
MAR	0.0066	0.0079	—	−0.0051	0.0095	0.0104	0.0166	0.0018	0.0084	0.0171		0.0008	0.0014	**0.0636**
BEQ	0.0049	0.0009	—	0.0067	0.0005	0.0005	−0.0050	−0.0078	−0.0030	−0.0025	0.0129		0.0009	**0.0592**
CAN	0.0113	−0.0025	—	0.0050	0.0098	0.0143	0.0132	−0.0004	0.0089	0.0044	0.0105	−0.0021		**0.0587**
BARB	0.0134	0.0251	—	0.0180	0.0008	0.0044	0.0046	−0.0031	−0.0041	0.0148	**0.0322**	0.0035	**0.0171**	

Isolation by distance of microsatellites in crab populations was detected by Mantel tests (*F*_ST_ vs. km), both for the whole dataset (*r* = .656; *p* < .00001) and within the Lesser Antilles (*r* = .503; *p* < .002). No isolation by distance was detected for sea urchin populations for the whole dataset (*r* = .068; *p* = .35) or within the Lesser Antilles (*r* = −.058; *p* = .63).

**Figure 3:**
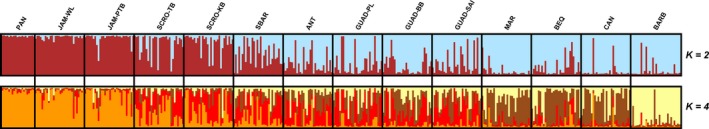
STRUCTURE bar plots for *K* = 2 (top), *K* = 4 (bottom) in *Dissodactylus primitivus*. Each line corresponds to an individual that was assigned with a certain probability to each genetic cluster

In *M. ventricosa*, STRUCTURE identified that the most probable number (*K*) of genetic clusters was one. In *D. primitivus*, the most probable *K* was four for the original method and two for the Evanno method. The bar plot for *K* = 2 (Figure 3) showed that most individuals from Panama Jamaica and St Croix were assigned to one genetic cluster. On the other hand, Martinique, Bequia, Canouan, and Barbados were highly associated with the other cluster, while those from remaining islands (St Barthélemy, Antigua, Guadeloupe) had more intermediate assignments. For *K* = 4 (Figure 3), the same situation was observed except that Barbados segregated in a single genetic cluster. The most probable *K* value in BAPS was 6, and the same subdivisions as those obtained with STRUCTURE were globally observed (Appendix S5).

divMigrate did not detect any asymmetric gene flow in *M. ventricosa*. In *D. primitivus*, several instances of asymmetric gene flow from Barbados to other islands (Guadeloupe, St Barthélémy, St Croix and Jamaica) were identified (Appendix S11).

## 4 DISCUSSION

### 4.1 Contrast between host and parasite and its potential causes

The two interacting species exhibit highly contrasting genetic structures within the Caribbean Sea. The genetic diversity of the parasitic crab *D. primitivus* is structured between two main groups. One is mostly found in the western part of the Caribbean and the other one, in the eastern part. On the contrary, the sea urchin host *M. ventricosa* exhibits no genetic structure (either in mitochondrial and nuclear markers) across the entire investigated geographic area.

Both species exhibited signs of population expansion but a more recent expansion or a larger population size for the sea urchin host might explain the sharp contrast between the two species. It is likely that there are different magnitudes of gene flow taking into account the dissimilar dispersal abilities of the two species. Whereas both adult crabs and sea urchins are able to move, such movements are very local (tens of meters). Therefore, dispersal capacity is related to pelagic larval stages. It is conventional to consider pelagic larval duration (PLD) as the main contributor to dispersal distance, although the relationship of PLD with genetic structure varies between species (Dawson, 2014; Faurby & Barber, 2012; Shanks, 2009; Shulman & Bermingham, 1995). The PLD of *M. ventricosa* is unknown while the one of *D. primitivus* is approximately 2 weeks (Pohle & Telford, 1983). Based on other tropical sea urchins with pluteus larvae (Emlet et al., 1987), it is probable that the PLD of *M. ventricosa* is at least equal to the one of *D. primitivus*. PLD might be one of the contrasting life history traits between these species but this need to be evaluated for *M. ventricosa*. At least three other factors might be linked to the contrasting dispersal abilities of *M. ventricosa* and *D. primitivus*. First, high fecundity has a positive influence on dispersal by increasing the number of potential migrants (Johnston, Miller, & Baums, 2012; Palumbi, 1994). *Dissodactylus primitivus* produces around 300 eggs per clutch (Jossart et al., 2014), whereas sea urchins with pluteus larvae produce millions of eggs per spawn (Emlet et al., 1987). Second, the larvae of the two species have different swimming capacities. While both sea urchin and crab larvae are reported to have behavioral mechanisms that might decrease dispersal, sea urchin larvae are weak swimmers that can be dispersed by currents far from the spawning location (Yednock & Neigel; Metaxas, 2013). Crab larvae are better swimmers, able to decrease drifting, which increases local recruitment (Yednock & Neigel, 2011). Third, there are differences in the availability and suitability of settlement habitats (Cowen & Sponaugle, 2009; Treml, Halpin, Urban, & Pratson, 2008). The distribution of sandy banks—favorable habitats for the sea urchin—can cover several square kilometers and are present in most areas of the Caribbean, allowing frequent settlement after long‐distance dispersal for *Meoma* larvae. The suitable area for recruitment is much more limited for crab larvae. They must find a habitat populated by the sea urchin, locate a host, and settle on its body (an area much smaller than the sand patches on which sea urchin larvae can settle).

If sharp differences in gene flow exist between these species, it would have implications for the evolution of parasitic interactions. Theoretical models (e.g., Blanquart, Kaltz, Nuismer, & Gandon, 2013; Gandon, 2002) and a meta‐analysis (Greischar & Koskella, 2007) suggest that when gene flow is higher in the host than in the parasite, natural selection will act on more alleles in the host which would adapt faster thus limiting the impact of its parasites. On the other hand, very high levels of gene flow may prevent local adaptation (Lenormand, 2002). The genetic homogeneity of *M. ventricosa* across the Caribbean Sea suggests such a lack of local adaptation potential. There are no data available for comparing parasitic success or infection consequences among populations of the pair *M. ventricosa*–*D. primitivus*. It would be interesting to acquire such data and compare them with those already obtained in Jamaica (De Bruyn et al., 2009, 2010).

### 4.2 Comparisons with other taxa and with proposed biogeographic regions in the Caribbean

Genetic homogeneity of *M. ventricosa* populations was observed across the Panamanian region and the Eastern Caribbean. This pattern was previously detected within the Caribbean in other taxonomic groups (Johnston et al., 2012 *;* Purcell et al., 2006; Silberman et al., 1994), as well as in other sea urchins (Lessios, Kane, & Robertson, 2003; Lessios, Kessing, & Pearse, 2001; Lessios et al., 2012; McCartney, Keller, & Lessios, 2000; Zigler & Lessios, 2004). The present study confirms this last observation, not only for mitochondrial DNA, but also for microsatellites.

The observed pattern of *D. primitivus* indicated that populations in Panama–Jamaica, but also to a lesser extent St. Croix, were differentiated from those in the other islands of the Lesser Antilles. This West‐East differentiation was suggested by predictive models (Cowen et al., 2006; Kool et al., 2010) and evidenced in several taxa (Andras et al., 2013; Baums, Miller, & Hellberg, 2005; DeBiasse et al., 2016; Diáz‐Ferguson et al., 2010; Foster et al., 2012; Purcell et al., 2006). In their model for defining biogeographic regions in the Caribbean, Cowen et al. (2006) nevertheless suggested that Jamaica represents a zone of mixing among the connectivity regions. Our data rather suggest that the mixing zone for this crab species is located at the North‐West of Lesser Antilles (St Croix—St Barthélemy islands).

### 4.3 Refinements in the genetic structure of the parasitic crab

The crab populations can be considered as genetically homogenous across several sets of locations. This is the case for all sites from the same islands, confirming previous results obtained for Jamaican coasts (Jossart et al., 2013). We also observed a genetic proximity (especially for COI) between Panama and Jamaica despite a large geographic distance. This can be explained by a recent range expansion event in the Western Caribbean (Appendix S6, star‐shaped haplotype network).

Within the Lesser Antilles, we identified heterogeneity in genetic variation of *D. primitivus*. First, as noted above, both COI and microsatellite data suggest that the samples from St Croix are closer to Panama and Jamaica samples than those from other islands of Lesser Antilles.

Second, microsatellite data showed that, whereas comparison between Martinique and the Grenadines yielded *F*_ST_ values not significantly different from zero, there was slight but significant differentiation between Martinique and Guadeloupe (*F*_ST_ = 0.0194–0.0328). This cannot be explained by geographical distance, because the distance between Martinique and the Grenadines (Bequia, Canouan) is comparable to the distance between Martinique and Guadeloupe. A potential explanation is linked to the different speeds of currents flowing between Guadeloupe and Martinique vs. Martinique and the Grenadines (Baums et al., 2005; Gyory et al., 2013; Figure 1). It is probable that *D. primitivus* can resist (or at least reduce) drifting in currents with speeds of several tens of centimeters per second, as it was demonstrated in other decapod larvae (Fernandez, Iribarne, & Armstrong, 1994; Luckenbach & Orth, 1992; Yednock & Neigel, 2011). However, in the zone South to Guadeloupe, the Caribbean east‐west current has higher speed, promoting high gene flow between these southern islands (Martinique, Grenadines).

Finally, we observed a moderate segregation of Barbados population (in both COI and microsatellite analyzes) despite its geographical proximity to other islands. Moreover, some cases of gene flow calculated between Barbados and its neighboring islands were asymmetric. This corroborates the study of Roberts (1997), who estimated that the larval import in coral reefs of Barbados is one of the lowest in the Caribbean. However, as genetic diversity of *D. primitivus* at this island is not lower than in other islands, an external input should be considered possibly from South America where *M. ventricosa* and *D. primitivus* are present (Wirtz et al., 2009).

### 4.4 Quaternary climatic oscillations

Our results suggest that Quaternary climatic oscillations (glacial–interglacial periods) (Miller et al., 2005; Peltier & Fairbanks, 2006) had an influence on the distribution of genetic diversity of crab and sea urchin populations. Several sea level falls of 100–150 m below the present level were related to the succession of glacial episodes (starting 2.7 Ma, more important 0.8 Ma when the glacial periods became much stronger, and ending with the last glacial maximum 0.02 Ma; Pillans & Gibbard, 2012). This temporal pattern coincides with both the expansions calculated from mismatch and EBSP analyzes. A similar population expansion was also suggested for two Caribbean squirrelfishes (Bowen, Bass, Muss, Carlin, & Robertson, 2006), for a corallivorous mollusk (Johnston et al., 2012), for a sea urchin (Lessios et al., 2003), and for a pea crab (Ocampo et al., 2013). This expansion might be linked to an increase in habitat availability during interglacial periods. Indeed, *M. ventricosa* is a coral‐associated organism that could have thrived with the coral reef expansion during these interglacial times (Baums, Scott Godwin, Franklin, Carlon, & Toonen, 2013; Bowen et al., 2006; Johnston et al., 2012). For the crab, population expansions were also suggested by mismatch analyzes. However, the large confidence intervals on the time estimates do not clarify whether these expansions happened synchronously everywhere and simultaneously with the host expansion.

## AUTHOR CONTRIBUTIONS

BD, CDR, HAL, QJ, RAW, TR conceived and designed the experiments; BD, CDR, HAL, QJ, SM, TR performed the sampling; MB and QJ performed the laboratory work; BD, CDR, HAL, QJ, RAW analyzed the data; BD, CDR, HAL, RAW, TR contributed reagents/materials/analysis tools; BD, CDR, HAL, MB, QJ, RAW, SM, TR wrote the manuscript.

## CONFLICT OF INTEREST

None declared.
