# Supplementary material for: Highly contrasted population genetic structures in a host–parasite pair in the Caribbean Sea
Source: Ecol Evol. 2017 Oct 4;7(22):9267–80. doi: 10.1002/ece3.3413 (PMC5696394; doi:10.1002/ece3.3413)

**SUPPLEMENTARY INFORMATION**

**Appendix S1.** Haplotype network for 652 bp COI analysis in *D. primitivus*. Some sites (undifferentiated, see results) from the same island were pooled to improve visualization. Sample size are indicated above the island’s abbreviation.


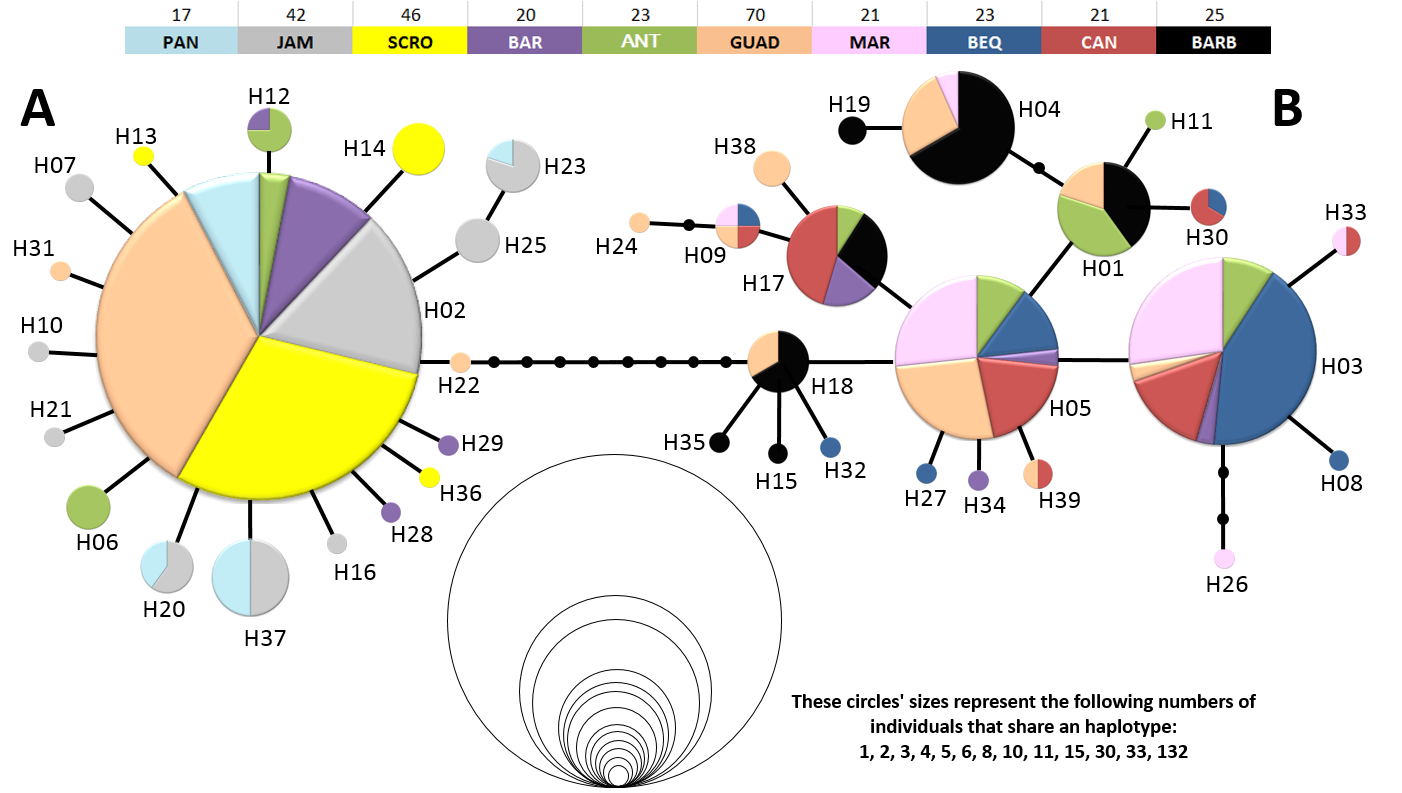


**Appendix S2.** Haplotype network for 758 bp COI analysis in *M. ventricosa*. Some sites (undifferentiated, see results) from the same island were pooled to improve visualization. Sample size are indicated next to the island’s abbreviation.


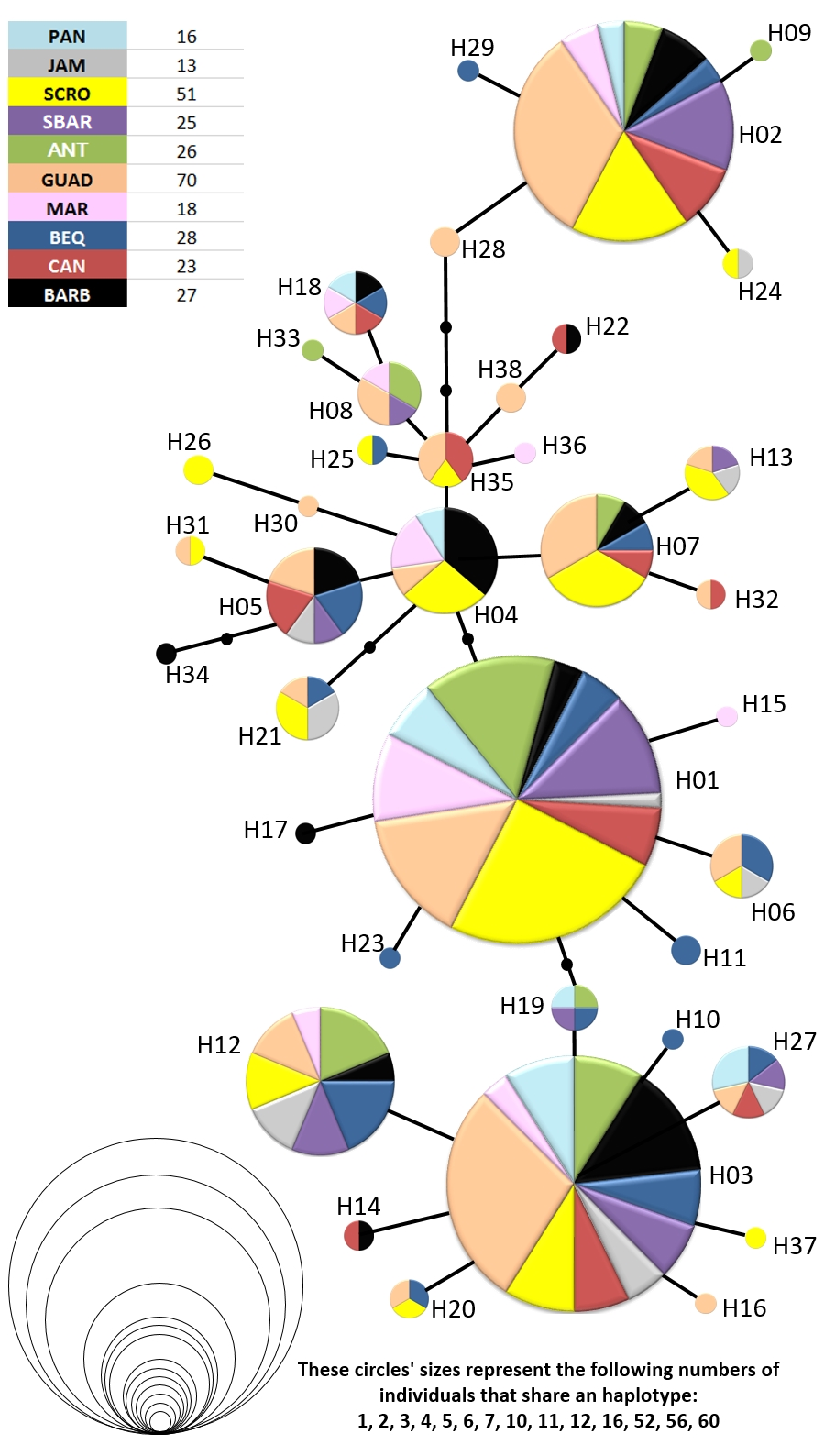


|  | **PAN** | **JAM-WL** | **JAM-PTB** | **SCRO-TB** | **SCRO-KB** | **SBAR** | **ANT** | **GUAD-PL** | **GUAD-BB** | **GUAD-SAI** | **MAR** | **BEQ** | **CAN** | **BARB** |
| --- | --- | --- | --- | --- | --- | --- | --- | --- | --- | --- | --- | --- | --- | --- |
| **C115** | -0.232 | 0.050 | -0.401 | -0.229 | 0.183 | -0.224 | -0.100 | -0.027 | 0.086 | 0.149 | 0.134 | -0.215 | -0.060 | -0.131 |
| **C118** | -0.017 | 0.008 | -0.057 | -0.063 | 0.049 | -0.038 | -0.058 | 0.049 | -0.073 | -0.088 | -0.054 | 0.055 | -0.055 | -0.064 |
| **A113** | -0.059 | -0.092 | -0.240 | 0.109 | 0.124 | -0.054 | -0.123 | 0.009 | -0.004 | -0.021 | -0.117 | -0.132 | 0.040 | -0.077 |
| **C4** | -0.208 | -0.109 | -0.096 | 0.040 | -0.028 | -0.201 | -0.248 | 0.065 | 0.099 | 0.100 | -0.261 | -0.126 | 0.054 | -0.118 |
| **A5** | -0.111 | -0.055 | -0.169 | -0.026 | 0.163 | -0.063 | -0.143 | 0.090 | -0.043 | -0.041 | -0.147 | 0.020 | 0.165 | 0.047 |
| **A101** | -0.003 | -0.095 | 0.005 | -0.071 | 0.041 | 0.032 | -0.054 | -0.119 | -0.105 | -0.153 | 0.022 | -0.070 | -0.027 | -0.141 |
| **D111** | -0.014 | 0.036 | 0.033 | 0.110 | 0.027 | -0.167 | 0.261 | -0.011 | 0.269 | 0.167 | 0.414 | 0.360 | 0.193 | 0.044 |
| **D110** | 0.152 | -0.155 | -0.024 | -0.057 | 0.005 | -0.157 | -0.041 | 0.092 | -0.087 | 0.002 | -0.026 | -0.239 | -0.137 | 0.003 |
| **C110** | -0.333 | -0.199 | -0.213 | 0.066 | 0.059 | -0.091 | -0.275 | -0.105 | -0.153 | -0.018 | -0.014 | -0.098 | -0.170 | -0.188 |
| **C9** | -0.360 | -0.183 | -0.217 | 0.279 | 0.012 | -0.074 | -0.093 | 0.230 | 0.146 | -0.120 | -0.077 | 0.030 | 0.187 | 0.143 |

**Appendix S3.** *F_IS_* values for each locus and population of *D. primitivus* (microsatellite data)**.**

**Appendix S4.** *F_IS_* values for each locus and population of *M. ventricosa* (microsatellite data).

|  | **PAN** | **JAM** | **SCRO-TB** | **SCRO-KB** | **SBAR** | **ANT** | **GUAD-PL** | **GUAD-BB** | **GUAD-SAI** | **MAR** | **BEQ** | **CAN** | **BARB** |
| --- | --- | --- | --- | --- | --- | --- | --- | --- | --- | --- | --- | --- | --- |
| **CHCM** | -0.028 | -0.078 | -0.016 | 0.011 | -0.062 | -0.089 | -0.026 | -0.080 | 0.019 | -0.079 | 0.027 | -0.031 | -0.076 |
| **9901** | 0.413 | 0.000 | 0.027 | -0.096 | -0.032 | -0.276 | 0.074 | -0.191 | -0.065 | -0.105 | 0.138 | -0.047 | 0.002 |
| **CHTO** | -0.132 | 0.126 | 0.323 | 0.154 | 0.136 | 0.023 | -0.018 | 0.038 | 0.162 | -0.088 | 0.069 | 0.083 | 0.034 |
| **93XX** | 0.239 | -0.102 | 0.124 | -0.129 | -0.002 | -0.205 | 0.069 | -0.014 | 0.036 | -0.129 | -0.081 | 0.022 | 0.237 |
| **NLQK** | 0.573 | 0.322 | 0.349 | 0.055 | 0.344 | 0.234 | 0.373 | 0.370 | 0.436 | 0.398 | 0.519 | 0.451 | 0.150 |
| **9U51** | -0.308 | -0.210 | 0.133 | 0.216 | 0.140 | 0.026 | 0.102 | -0.197 | -0.081 | -0.128 | 0.053 | 0.146 | 0.137 |
| **6SKB** | 0.223 | 0.743 | 0.492 | 0.503 | 0.686 | 0.626 | 0.610 | 0.325 | 0.517 | 0.416 | 0.624 | 0.634 | 0.759 |
| **TCYO** | -0.056 | -0.065 | -0.007 | 0.338 | -0.007 | -0.035 | 0.006 | 0.178 | -0.104 | 0.269 | 0.196 | 0.040 | 0.373 |

**Appendix S5.** BAPS bar plots for K = 6 in *D. primitivus*. Each line corresponds to an individual that was assigned with a certain probability to each genetic cluster.


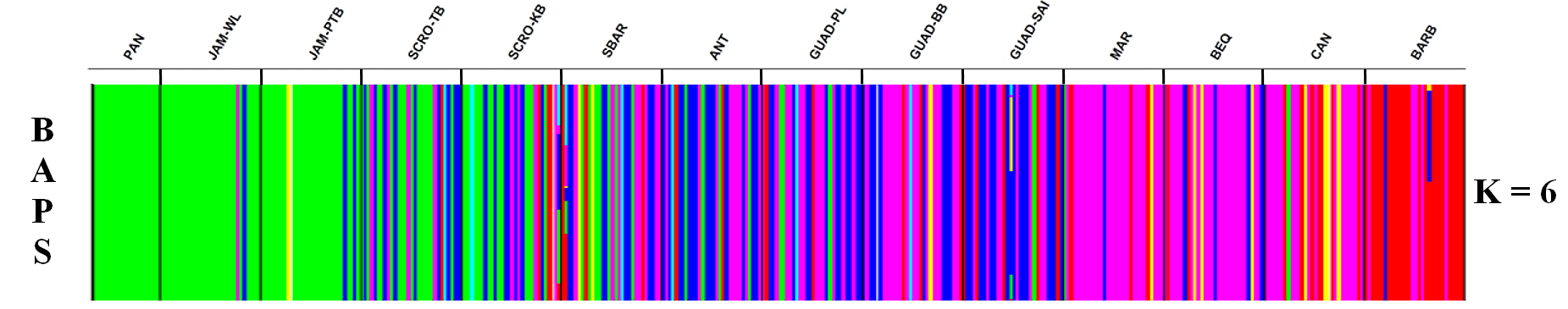


|  |  | **Fu’s *F_S_*** | | | **Mismatch distribution analysis** | | | | | | | | | | | |
| --- | --- | --- | --- | --- | --- | --- | --- | --- | --- | --- | --- | --- | --- | --- | --- | --- |
|  |  |  |  |  | **Demographic expansion** | | | **Spatial expansion** | | | | | | | | |
|  |  | ***F_S_*** | **P** | **↑→** | **SSD** | **P** | **↑→** | **SSD** | **P** | **↑→** | **τ** | **τ min** | **τ max** | **t** | **t min** | **t max** |
| *Mv* | All | -18.49 | 0.0008 | **↑** | 0.0331 | 0.012 | **→** | 0.0275 | 0.066 | **↑** | 3.93 | 1.89 | 5.28 | 341 509 | 164 455 | 459 155 |
|  |  |  |  |  |  |  |  |  |  |  |  |  |  |  |  |  |
| *Dp* | PAN | -0.58 | 0.29 | **→** | 0.0072 | 0.428 | **↑** | 0.0072 | 0.347 | **↑** | 0.92 | 0.41 | 1.79 | 70 623 | 31 209 | 137 334 |
|  | JAM | -4.31 | 0.008 | **↑** | 0.0053 | 0.297 | **↑** | 0.0053 | 0.177 | **↑** | 1.17 | 0.56 | 1.68 | 89 555 | 42 620 | 128 491 |
|  | SCRO | -2.19 | 0.033 | **→** | 0.0060 | 0.406 | **↑** | 0.0016 | 0.122 | **↑** | 0.33 | 0.10 | 0.86 | 25 503 | 7 561 | 66 323 |
|  | SBAR | 0.78 | 0.67 | **→** | 0.1044 | 0.204 | **↑** | 0.0595 | 0.257 | **↑** | 11.99 | 0.36 | 90.37 | 919 688 | 27 544 | 6 930 094 |
|  | ANT+GUAD | 0.93 | 0.68 | **→** | 0.0769 | 0.188 | **↑** | 0.0212 | 0.691 | **↑** | 11.57 | 1.21 | 18.41 | 887 373 | 92 797 | 1 411 694 |
|  | MAR+BEQ+CAN | -5.23 | 0.009 | **↑** | 0.0037 | 0.486 | **↑** | 0.0031 | 0.379 | **↑** | 0.87 | 0.57 | 2.48 | 67 012 | 43 396 | 190 117 |
|  | BARB | -0.16 | 0.50 | **→** | 0.0366 | 0.187 | **↑** | 0.0308 | 0.338 | **↑** | 3.11 | 0.84 | 5.16 | 238 597 | 64 248 | 395 389 |

**Appendix S6.** Fu’s *F_S_* statistic and mismatch distribution analysis based on COI data. SSD: sum of squared deviation. A vertical arrow indicates expansion while a horizontal arrow indicates stability. For *Dp,* alpha was Benjamini-Yekutieli corrected to 0.019. **τ**: expansion time parameter, t: time of expansion.

**Appendix S7.** Extended Bayesian Skyline Plot (EBSP) for the sea urchin *M. ventricosa* (CO1 sequences). The x-axis indicates the time in million years and the y axis is the effective population size (logarithmic scale)


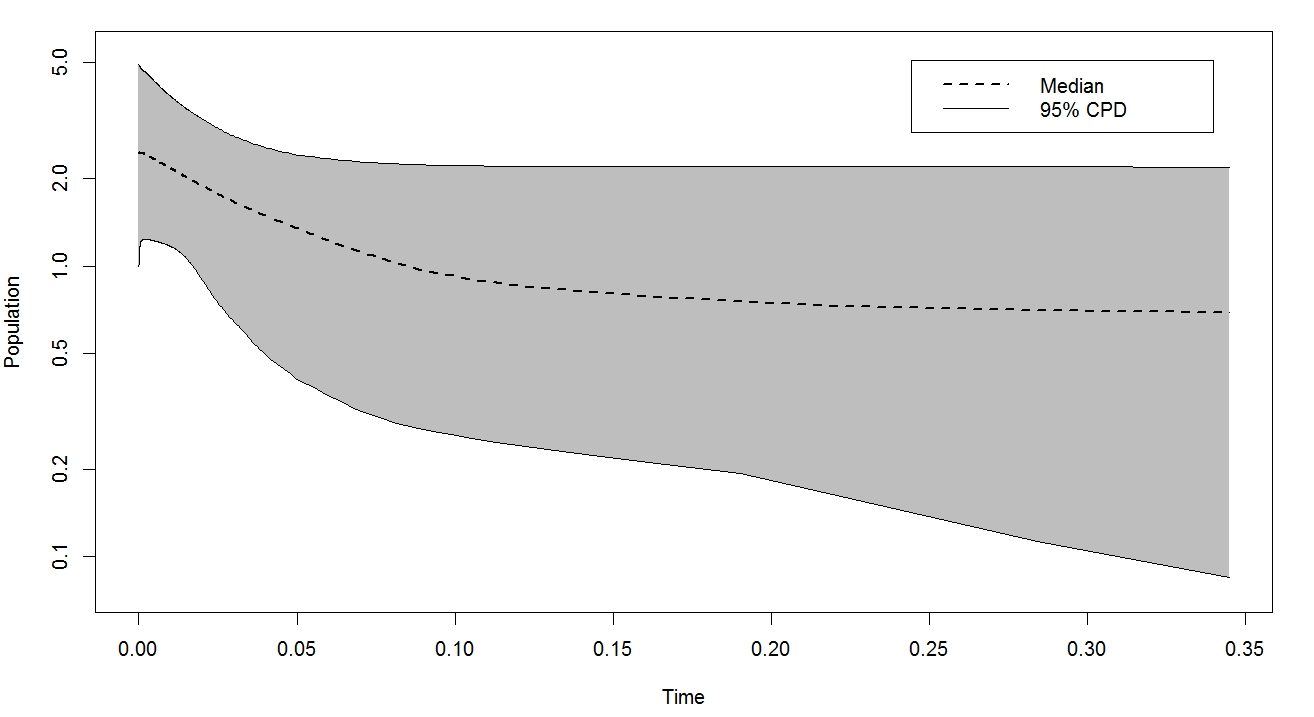


**Appendix S8.** Pairwise *F_ST_* values (Weir & Cockerham 1984) between populations for COI analysis in *D. primitivus* (above diagonal) and *M. ventricosa* (below diagonal). Bold values differ significantly from zero (based on 10,000 permutations). The alpha was corrected by Benjamini-Yekutieli method (Narum 2006) and was 0.0094 for *D. primitivus* and 0.0099 for *M. ventricosa*.

|  | **PAN** | **JAM-WL** | **JAM-PTB** | **SCRO-TB** | **SCRO-KB** | **SBAR** | **ANT** | **GUAD-PL** | **GUAD-BB** | **GUAD-SAI** | **MAR** | **BEQ** | **CAN** | **BARB** |
| --- | --- | --- | --- | --- | --- | --- | --- | --- | --- | --- | --- | --- | --- | --- |
| **PAN** |  | -0.03533 | -0.00108 | 0.13276 | 0.10257 | 0.02224 | **0.15269** | 0.03923 | 0.04943 | 0.03132 | **0.34179** | **0.38291** | **0.27281** | **0.28648** |
| **JAM-WL** | -0.00196 |  | -0.02868 | **0.11607** | 0.08939 | 0.00711 | **0.12579** | 0.02249 | 0.03819 | 0.01652 | **0.30826** | **0.34788** | **0.24209** | **0.25636** |
| **JAM-PTB** | 0.00752 | 0.02225 |  | **0.12982** | **0.10197** | 0.00376 | **0.10048** | 0.01675 | 0.04402 | 0.01424 | **0.27893** | **0.31984** | **0.21173** | **0.22714** |
| **SCRO-TB** | -0.01154 | -0.02463 | 0.04856 |  | -0.03161 | 0.07550 | **0.32583** | 0.10855 | 0.02467 | 0.07643 | **0.53215** | **0.56573** | **0.46599** | **0.46888** |
| **SCRO-KB** | 0.02363 | -0.02114 | 0.02416 | -0.00773 |  | 0.04900 | **0.30030** | 0.08245 | 0.00352 | 0.05045 | **0.50581** | **0.53970** | **0.44062** | **0.44514** |
| **SBAR** | -0.00679 | -0.00869 | 0.03932 | -0.01572 | -0.00428 |  | **0.11432** | -0.00549 | -0.00414 | -0.01119 | **0.30014** | **0.34275** | **0.22246** | **0.26631** |
| **ANT** | -0.00810 | -0.02541 | 0.02990 | -0.01785 | 0.00464 | -0.01288 |  | **0.10649** | **0.20295** | **0.14170** | **0.11097** | **0.15923** | 0.06531 | **0.12658** |
| **GUAD-PL** | 0.00475 | 0.06283 | 0.01076 | 0.04390 | **0.05747** | 0.03577 | 0.04447 |  | 0.00883 | -0.02236 | **0.26917** | **0.33971** | **0.21585** | **0.23694** |
| **GUAD-BB** | -0.00628 | 0.00534 | 0.01859 | -0.00982 | -0.00190 | -0.01235 | 0.01368 | 0.00126 |  | -0.01338 | **0.39485** | **0.43788** | **0.33624** | **0.35976** |
| **GUAD-SAI** | 0.00973 | 0.01222 | 0.04382 | 0.00684 | 0.00282 | -0.02439 | 0.01833 | 0.03325 | -0.01658 |  | **0.31576** | **0.37285** | **0.25666** | **0.25794** |
| **MAR** | -0.01059 | -0.05181 | 0.04097 | -0.02714 | -0.01869 | -0.02060 | -0.02346 | 0.05409 | 0.00022 | -0.00157 |  | 0.02250 | 0.02890 | **0.23814** |
| **BEQ** | 0.00584 | 0.00444 | -0.02608 | 0.02431 | 0.00805 | 0.01684 | 0.01599 | 0.01421 | 0.00033 | 0.02094 | 0.01514 |  | 0.09695 | **0.29200** |
| **CAN** | -0.01292 | -0.00540 | 0.01766 | -0.00885 | 0.00378 | -0.01564 | 0.00996 | 0.00380 | -0.02603 | -0.01833 | -0.00381 | -0.00160 |  | **0.16383** |
| **BARB** | -0.00785 | 0.01941 | 0.00834 | 0.03149 | 0.03876 | 0.02923 | 0.04014 | -0.00840 | -0.00417 | 0.02509 | 0.02472 | 0.00883 | -0.00243 |  |

**Appendix S9.** Analyses of molecular variance (AMOVA) for COI (left) and microsatellites (right). Significances of Φ or *F* values were determined by permutation tests (10,000 randomizations). Regions were defined according to the SAMOVA analysis: Region 1 (Panama, Jamaica, St Croix, Saint Barthélemy, Antigua and Guadeloupe), Region 2 (Martinica, Bequia, Canouan and Barbados).

| ***D. primitivus*** | **CO1** | | **P** | **Variance** | **% of variation** | **Microsatellites** | | **P** | **Variance** | **% of variation** |
| --- | --- | --- | --- | --- | --- | --- | --- | --- | --- | --- |
| **Among regions** | ***Φ_CT_*** | 0.666 | <0.001 | 3.65 | 66.6 | ***F_CT_*** | 0.0261 | <0.0001 | 0.109 | 3 |
| **Among populations within regions** | ***Φ_SC_*** | 0.205 | <0.001 | 0.38 | 6.9 | ***F_SC_*** | 0.0285 | <0.0001 | 0.116 | 3 |
| **Within populations** | ***Φ_ST_*** | 0.734 | <0.001 | 1.45 | 26.5 | ***F_ST_*** | 0.054 | <0.0001 | 3.954 | 97 |
|  |  |  |  |  |  |  |  |  |  |  |
| ***M. ventricosa*** | **CO1** | | **P** | **Variance** | **% of variation** | **Microsatellites** | | **P** | **Variance** | **% of variation** |
| **Among regions** | ***Φ_CT_*** | -0.001 | 0.53 | 0 | 0 | ***F_CT_*** | 0.00001 | 0.42 | 0.0003 | 0.01 |
| **Among populations within regions** | ***Φ_SC_*** | 0.005 | 0.23 | 0.01 | 0.5 | ***F_SC_*** | 0.008 | <0.001 | 0.026 | 1 |
| **Within populations** | ***Φ_ST_*** | 0.004 | 0.26 | 1.62 | 99.5 | ***F_ST_*** | 0.008 | <0.001 | 3.129 | 98.99 |

**Appendix S10.** ΦST vs km for COI in *D. primitivus* (reporting r and P values of the Mantel tests)*.* The orange box corresponds to the whole data set and the green box to the connectivity region within the Lesser Antilles defined by Kool et al (2010) (see Figure 1).


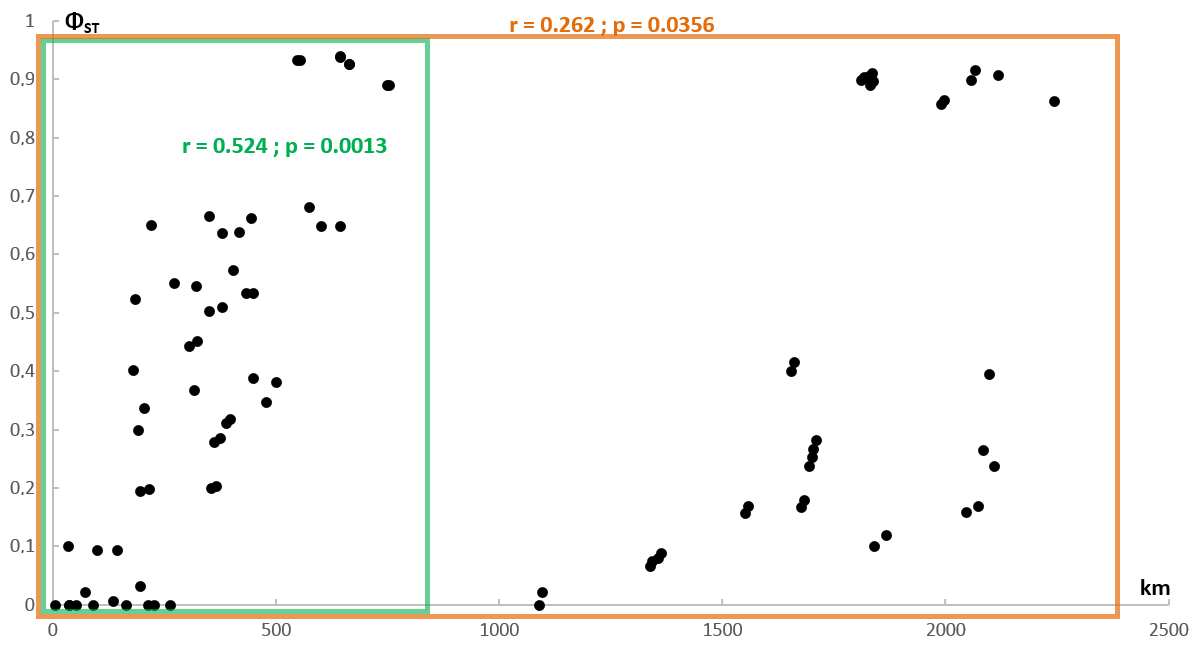


**Appendix S11.** Directional relative migration network for crab populations (divMigrate output). Arrows and relative migration values are only represented if the migration was significantly asymmetric between a pair of populations.


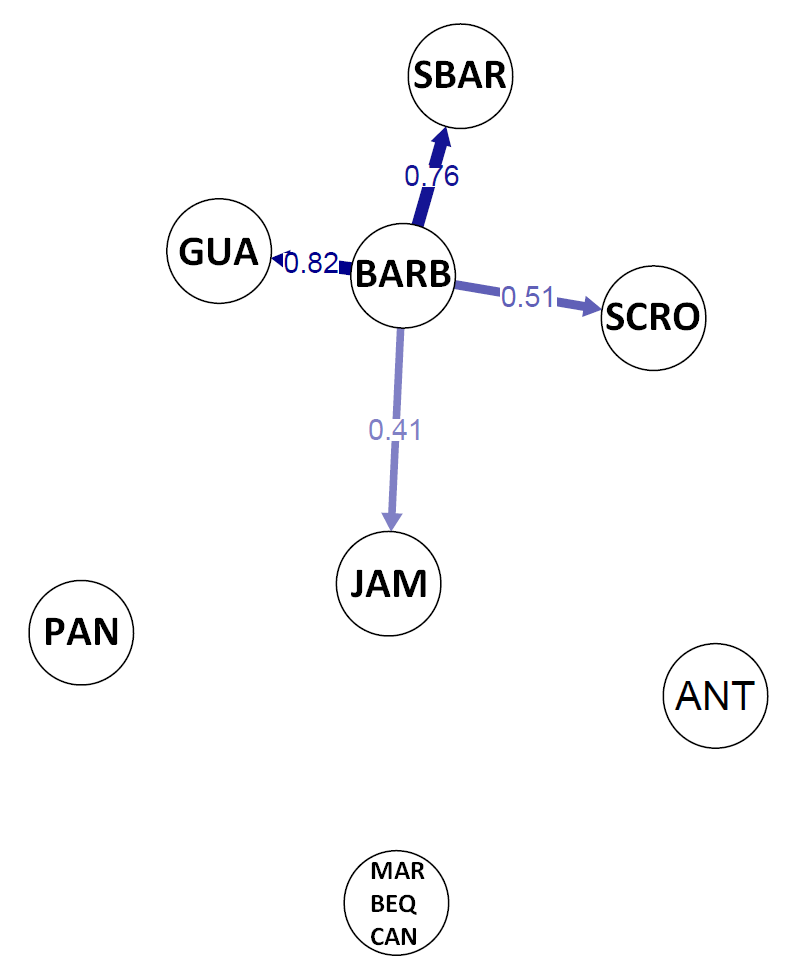

Supplement: Supplementary file 1 [file ECE3-7-9267-s001.docx]
